# Vein of Galen aneurysmal malformation in newborns: a retrospective study to describe a paradigm of treatment and identify risk factors of adverse outcome in a referral center

**DOI:** 10.3389/fped.2023.1193738

**Published:** 2023-07-20

**Authors:** Silvia Buratti, Marisa Mallamaci, Giulia Tuo, Mariasavina Severino, Domenico Tortora, Costanza Parodi, Andrea Rossi, Francesco Pasetti, Lucio Castellan, Valeria Capra, Ferruccio Romano, Patrizia De Marco, Marco Pavanello, Gianluca Piatelli, Dario Paladini, Maria Grazia Calevo, Andrea Moscatelli

**Affiliations:** ^1^ Neonatal and Pediatric Intensive Care Unit, Acceptance and Emergency Department, IRCCS Istituto Giannina Gaslini, Genoa, Italy; ^2^ Department of Neuroscience (DINOGMI), University of Genoa, Genoa, Italy; ^3^ Pediatric Cardiology and Cardiac Surgery Unit, Surgery Department, IRCCS Istituto Giannina Gaslini, Genoa, Italy; ^4^ Neuroradiology Unit, Services Department, IRCCS Istituto Giannina Gaslini, Genoa, Italy; ^5^ Department of Health Sciences (DISSAL), University of Genoa, Genoa, Italy; ^6^ Pediatric Radiology Unit, Services Department, IRCCS Istituto Giannina Gaslini, Genoa, Italy; ^7^ Neuroradiology Unit, Diagnostic Imaging and Radiotherapy Department, San Martino Polyclinic Hospital, Genoa, Italy; ^8^ Genomics and Clinical Genetics Unit, IRCCS Istituto Giannina Gaslini, Genoa, Italy; ^9^ Medical Genetics Unit, IRCCS Istituto Giannina Gaslini, Genoa, Italy; ^10^ Neurosurgery Unit, Surgery Department, IRCCS Istituto Giannina Gaslini, Genoa, Italy; ^11^ Fetal Medicine and Surgery Unit, Department Mother and Child, IRCCS Istituto Giannina Gaslini, Genova, Italy; ^12^ Epidemiology, Biostatistics Unit, Scientific Direction, IRCCS Istituto Giannina Gaslini, Genova, Italy

**Keywords:** newborn, VGAM, heart failure, brain MR, echocardiography, outcome

## Abstract

**Background:**

Vein of Galen aneurysmal malformation (VGAM) is a rare cerebral vascular malformation associated with significant morbidity and mortality. Newborns with VGAM without adequate treatment may develop rapidly deteriorating high output heart failure (HOHF) and are at risk for severe neurological outcomes.

**Objective:**

To describe the clinical course and management of newborns with VGAM, and identify which echocardiographic and neuroradiologic factors may be associated with severe heart failure at birth and adverse short term outcomes.

**Methods:**

This is a single center retrospective cohort study including all consecutive newborns with VGAM admitted to Gaslini Children's Hospital between 2009 and 2022. We reviewed clinical data, intensive care support, fetal and neonatal cardiologic and neuroradiologic findings and we studied the association with severe HOHF, endovascular complications and death.

**Results:**

Out of 40 newborns, 17 (42.5%) developed severe HOHF requiring early endovascular procedures. Medical treatment was focused on the main components of HOHF by providing inotropic support and peripheral vasodilation. Pulmonary vasodilators were avoided to reduce the negative effects of pulmonary overflow and prevent vascular remodeling. Reduction of the obligatory left to right shunt through the VGAM was possible only through endovascular treatment. Fetal cardiothoracic ratio was significantly associated with severe HOHF at birth and death. Cardiologic parameters of right ventricular overload, pulmonary hypertension and systemic steal were the leading findings associated with haemodynamic compromise at birth. The mediolateral diameter of the straight or falcine sinus at its shortest section (SS-MD), and arterial pseudofeeders were significantly associated with severe HOHF at birth in prenatal and postnatal assessments. None of the postnatal echocardiographic and MRI variables, nor a higher inotropic support were associated with major periprocedural complications or death. Mortality was due to palliation for congenital severe brain damage (4/40, 10%), or major periprocedural complications (3/40, 7.5%). None of the patients died due to HOHF and multiorgan failure. Overall survival at discharge was 82.5% (33/40).

**Conclusions:**

The complexity of neonatal VGAM pathophysiology requires a multidisciplinary approach, specialized intensive care management, and early endovascular treatment to reduce mortality and optimize clinical outcomes. Cardiologic and neuroradiologic parameters are key to define risk stratification and treatment strategies.

## 1. Introduction

Vein of Galen aneurysmal malformation (VGAM) is a high flow-low resistance arteriovenous malformation of the choroidal arterial system. VGAM embryologically results from abnormal connections between the primitive choroidal vessels and the median prosencephalic vein of Markowski (1). It represents less than 1% of pediatric malformations, but it is the most frequent intracranial arteriovenous malformation in children. Incidence is about 1 out of 25,000 deliveries (2). A genetic background has been recently identified in VGAM patients, including mutations of *RASA1* (RASp21 Protein Activator 1) and *EPHB4* (Ephrin type-B receptor 4), genes encoding proteins involved in vascular development (3–5). Morbidity and mortality rates are still high despite progressive improvement in pathophysiological understanding of the disease and in treatment strategies. The diagnostic and therapeutic pathways are complex and based on a multidisciplinary approach. In the last three decades, endovascular treatment (EVT) and highly specialized management in neonatal and pediatric intensive care contributed to making VGAM a potentially treatable disease (6, 7).

During fetal life, the low-resistance placenta competes with the VGAM and diminishes the flow through the malformation. At birth, when the placental circulation is removed, the flow through the VGAM increases significantly and rapidly deteriorating respiratory and high-output heart failure (HOHF) occur in more than 50% of the patients (8, 9). This condition may lead to severe pulmonary hypertension and multiorgan failure, if not adequately treated. In less severe cases, newborns can be managed with medical treatment and the endovascular procedure can be deferred to 4–6 months of life. Relevant aspects of intensive care support and indication to palliation in newborns are still controversial.

In the last decades, several fetal and neonatal vascular and anatomic features have been studied to identify predictors of clinical severity and parameters for risk stratification of newborns with VGAM. Recently, Arko et al. identified a promising neuroimaging marker highly predictive of the *neonatal-at-risk* cohort, i.e., the mediolateral diameter of the straight or falcine sinus at its shortest section (SS-MD) (9). Moreover, the presence of fetal and neonatal arterial pseudofeeders has been associated with brain damage. Arterial pseudofeeders are dilated branches of the middle cerebral artery visible in the sylvian valley, thus they are not part of the choroidal system. They are interpreted as a sign of severe arterial steal, venous hypertension and overall cerebral blood flow impairment (10).

The aim of this study is to focus on the management of severe forms of VGAM that present with HOHF in the neonatal period and identify which cardiologic and neuroradiologic factors or variables may be associated with adverse outcomes in the neonatal period.

## 2. Materials and methods

### 2.1. Study design and aims

The primary purpose of this single-center retrospective cohort study is to identify which factors may be associated with clinical severity in the neonatal period.

Therefore, we analyzed the associations between:

- fetal echocardiographic and neuroradiologic findings with severe HOHF requiring EVT in the neonatal period;
- neonatal echocardiographic and neuroradiologic findings with severe HOHF requiring EVT in the neonatal period.

As secondary aim, we studied which fetal and neonatal echocardiographic and neuroradiologic factors may be associated with major periprocedural complications and mortality.

The rational and principles of intensive care management and strategies in our center are described.

Long term outcomes in this cohort are also briefly presented.

The study has been approved by the Regional Ethical Committee (CER Liguria: 804/2021). We used the STROBE checklist while writing this observational study (www.strobe-statement.org).

### 2.2. Setting

Gaslini Children's Hospital is a major pediatric tertiary care center where high-risk pregnancies are centralized. Our VGAM team is a multidisciplinary group that includes specialists in Genetics, Perinatal Medicine, Fetal and Pediatric Cardiology, Neurology, Neonatal and Pediatric Intensive Care, Interventional Radiology, Neuroradiology, and Neurosurgery. The Neonatal and Pediatric Intensive Care Unit admits every year about 100 high-risk newborns. Pregnant women carrying fetuses with VGAM follow a defined clinical pathway. Antenatal diagnosis is most often made during the third trimester ultrasound scan, less often in the second trimester (2). VGAM features, onset and progression of cerebral and/or haemodynamic complications are monitored by means of fetal ultrasound, brain MRI and serial echocardiography. Prenatal counseling is performed by the VGAM team. C-section is scheduled at term, or preterm if signs of rapidly deteriorating cardiac failure or neuroradiologic complications are demonstrated. Echocardiography and brain MRI are performed in the first 24–48 h of life and after each endovascular procedure.

### 2.3. Patients

We included all consecutive patients with a diagnosis of VGAM admitted in the neonatal period (<30 days of life) between January 2009 and June 2022, with follow-up to December 2022. Criteria to enroll patients were presence of clinical and radiological data obtained from fetal assessment, and postnatal cardiac US and brain MRI imaging. We collected the following data: prenatal evaluations (fetal echocardiography and MRI), birth weight and gestational age (GA), Apgar score, delivery room assistance, clinical course in the ICU, endovascular procedures and complications, echocardiography, neonatal brain MRI, and mortality. Exclusion criteria were: (i) age at presentation >30 days, (ii) absence of clinical information and/or low-quality/insufficient brain and cardiac imaging.

### 2.4. Prenatal assessment

#### 2.4.1. Prenatal cardiac ultrasound

Fetal echocardiogram was performed by a transabdominal approach using a pulsed, continuous, and color Doppler ultrasound system (Philips iE33). All cases received at least one detailed ultrasound scan that was performed in accordance with published standards (11). Gestational age was determined by last menstruation. The most recent echocardiogram before delivery was used for the statistical analysis. Fetal echocardiogram was aimed at assessment of cardiac overload after having ruled out the possible association with a congenital heart disease (12). The following two-dimensional echocardiographic data were retrieved: presence or absence of hydrops (effusion, ascites and/or skin edema), cardiothoracic ratio, subjective appearance of the superior vena cava (SVC), normal size or dilated. The cardiothoracic ratio, measured as cardiac area/thoracic area on a 4-chamber axial view of the fetal thorax, was considered abnormal if >0.50 (13). Color-flow Doppler was utilized to assess the degree of tricuspid regurgitation, which was qualitatively graded as absent, mild, moderate or severe based on jet width and length, and to assess the blood flow across the aortic isthmus (antegrade or reversal diastolic flow). Pulsed-wave Doppler was used for the evaluation of flow patterns in the ductus venosus (normal or reversal flow) (13–15).

#### 2.4.2. Fetal brain MRI

Fetal MRI studies were performed on a 1.5 T scanner with protocols including T2-weighted images on the three planes, axial T1-weighted images, and axial diffusion weighted images. The following parameters were recorded: orthogonal diameters of the VGAM (craniocaudal, laterolateral and anteroposterior), volume of the VGAM calculated using the ellipsoid formula, presence/absence of ventriculomegaly (defined as width of the ventricular atrium >9.9 mm), maximal mediolateral diameter of the straight or falcine sinus at its narrowest point in the craniocaudal axis (9), presence of arterial pseudofeeders (10), brain damage (including ischemic or hemorrhagic cerebral infarcts, white matter lesions, and/or global atrophy), and cerebral aqueduct compression. The most recent fetal MRI before delivery was used for the statistical analysis.

### 2.5. Postnatal assessment

#### 2.5.1. Postnatal cardiac ultrasound and cardiologic evaluation

**Figure 1:**
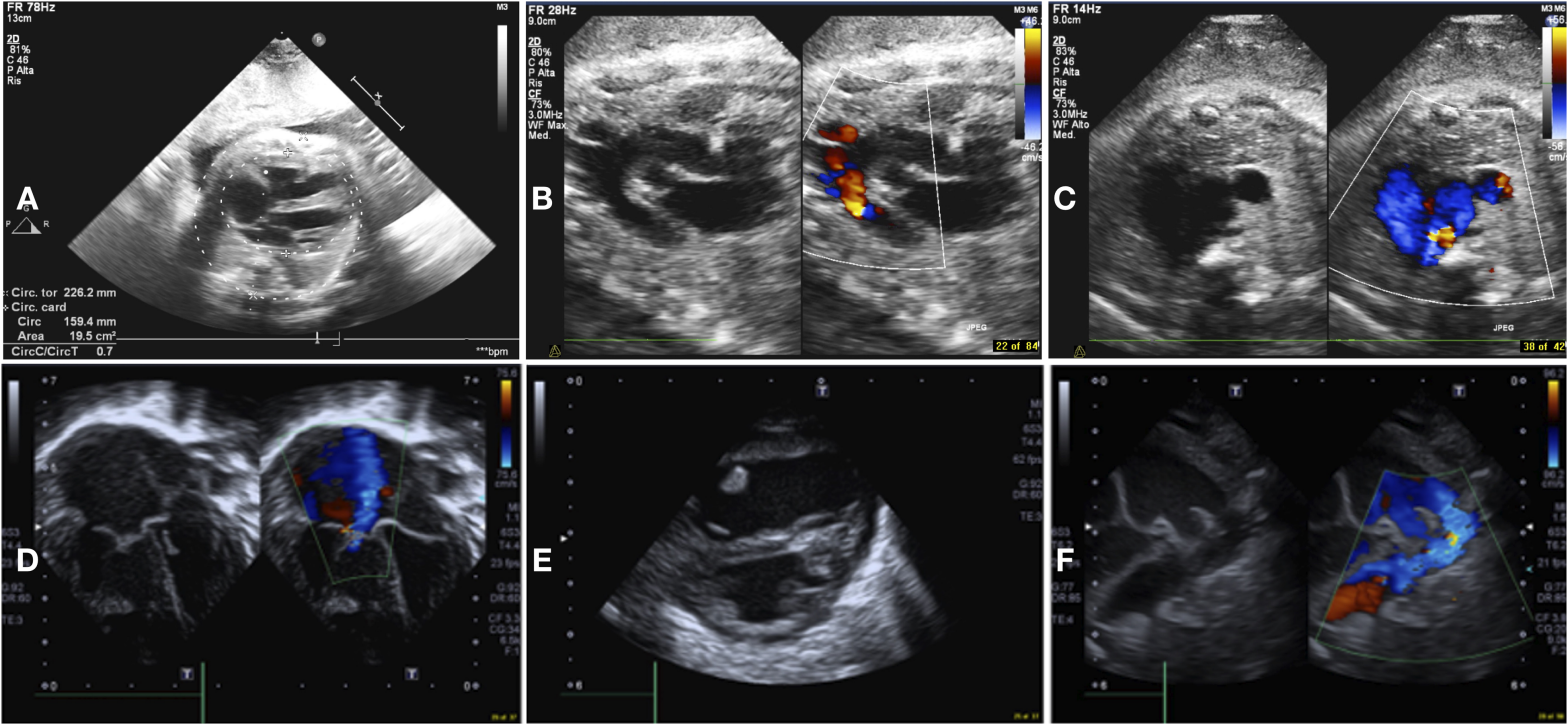
Fetal and neonatal echocardiographic images. Fetal images: (**A**) increased cardiothoracic index, 4-chamber axial view; (**B**) retrograde flow in aortic arch, long axis view; (**C**) superior vena cava dilation, 3 vessels view. Neonatal images: (**D**) right heart dilatation and tricuspid regurgitation, apical 4 chamber view; (**E**) flattening of the interventricular septum, parasternal short axis view; (**F**) right to left shunt at the level of the ductus arteriosus, standard “three-finger” ductal view.

Cardiovascular examination and echocardiogram were performed on every neonate on day of life 1 or immediately after the admission and were repeated at least before and after every endovascular procedure. The following echocardiographic parameters were reviewed: right end-diastolic diameters (REDD), left ventricular fractional shortening (LV-FS), estimation of pulmonary hypertension (PH) by means of PH index (ratio between systolic pulmonary artery pressure and systemic arterial pressure) and of flattening of the interventricular septum (IVS). The shape of the IVS was described, based on the right-to-left side motion, as normal, intermediate, and complete right-to-left shift with left ventricular collapse (16). Color and pulsed Doppler techniques were used to assess ductus arteriosus (DA) size and blood flow direction and reversal flow at the level of the aortic isthmus. Fetal and neonatal echocardiographic images are shown in Figure 1.

The echocardiographic evaluation performed before the first neonatal endovascular treatment was retrieved for the statistical analysis. This time point should represent the best hemodynamic status that we were able to reach implementing maximal medical treatment to further validate the indication to treatment.

All echocardiographic data were retrieved from our Cardiology Department database.

#### 2.5.2. Postnatal neuroimaging studies

Postnatal brain MRI studies were performed in every neonate on day of life 1 or at hospital admission and were usually repeated before each endovascular procedure. To detect periprocedural complications, brain MRI and/or CT scan were always performed shortly after the endovascular treatment.

Brain MRI studies were performed on a 1.5 T or 3 T scanner with dedicated protocols including: T2-weighted images on the three planes, 2D or 3D T1-weighted images, axial DWI, susceptibility weighted imaging, and arterial spin labeling perfusion. Arterial and venous MR angiography were always performed with 3D time-of-flight or phase-contrast techniques, respectively.

In addition to the prenatal neuroimaging features, in the first postnatal study we evaluated the type of VGAM and obtained data regarding the presence of superior sagittal sinus (SSS) stenosis, jugular bulb (JB) stenosis, internal cerebral vein drainage into the VGAM, and small thalamic feeders. The SSS index was also calculated as previously described (17) (i.e., width of the flow void in the sagittal sinus on coronal T2 spin-echo/biparietal diameter * 100).

All brain MRI studies were reviewed in consensus by two experienced pediatric neuroradiologists (MS and AR). All measurements were performed in consensus by a pediatric neuroradiologist (MS) and a bioinformatician (CP).

### 2.6. Intervention: medical therapy and endovascular treatment

Medical treatment was focused on the main components of HOHF by providing inotropic support and peripheral vasodilation. Pulmonary vasodilators were always avoided to reduce the negative effects of pulmonary overflow and prevent vascular remodeling. Epinephrine, milrinone and levosimendan were the vasoactive medications used. Vasoactive support was modulated to optimize end-organ perfusion, evaluated through lactate levels and urinary output.

Prostaglandin E_1_ infusion was indicated to maintain the DA open if severe right ventricular overload was present (PH index >1) (18). Diuretics were used to reduce pre-load. Non-invasive or invasive ventilation were often required to reduce cardiac workload. We recorded data about medical therapy, haemodynamic and respiratory support for patients with HOHF with dosage of vasoactive drugs. Inotrope score (IS) and vasoactive inotrope score (VIS) were calculated according to Gaies et al. (19): IS = dopamine dose [μg/kg/minute] + dobutamine dose [μg/kg/minute] + 100 × epinephrine dose [μg/kg/minute], VIS = IS + 10 × milrinone dose [mcg/kg/minute] + 10,000 × vasopressin dose [units/kg/minute] + 100 × norepinephrine dose [mcg/kg/minute]. The VIS has been proposed as a predictor of clinical outcomes based on severity (20).

The aim of the endovascular procedure was to reduce the effects of the AV shunt on cardiac function by occlusion of pathological arterial feeders of the vascular malformation. The endovascular procedure consisted in the deposition of n-butyl cyanoacrylate, other liquid embolic systems, like Onyx (Medtronic, Irvine, CA, USA), Squid (Balt, Montmorency, France) and PHIL (MicroVention, Aliso Viejo, CA, USA), or coils into distal VGAM feeders, performed by femoral artery catheterization, using a 1.2–1.5 French diameter microcatheter. Under fluoroscopic guidance, different concentrations of liquid embolic systems were injected depending on the AV flow, to reduce the risk of migration into the venous system.

Indication to endovascular procedure in the neonatal period was either severe HFOF refractory to medical treatment, or the presence of neuroradiological risk factors, such as arterial pseudofeeders, white matter lesions, ischemic infarcts, superior sagittal sinus stenosis, or jugular bulb stenosis, in the absence of severe HOHF.

The evidence of severe brain damage was the main contraindication to endovascular treatment and led to palliation. We never considered the presence of severe HOHF a contraindication to the procedure, even if maximal support was in place, since medical management was usually suited to prevent multiorgan failure.

### 2.7. Main outcome measures

As primary outcome we considered severe HOHF requiring endovascular treatment in the neonatal period. HOHF was defined by the presence of tachycardia, signs of respiratory distress, clinical and biochemical markers of poor organ perfusion (oliguria and lactic acidosis). Longitudinal clinical observation after birth and progressive implementation of medical therapy and respiratory support led to the definition of HOHF. Severe HOHF was defined by need of invasive ventilation and inotropic support to maintain gas exchange and organ perfusion.

As secondary outcomes we selected short term outcomes, including:

- occurrence and severity of periprocedural neurologic complications (hemorrhagic and/or ischemic lesions occurring within 1 week after endovascular procedure):
- mortality.

Minor periprocedural complications were defined as ischemic and/or hemorrhagic lesions with no or mild signs of neurological dysfunction. Major complications were defined as lesions associated with seizures and/or major neurological dysfunction. Causes of death were reported as: endovascular procedure complication, HOHF and multiorgan failure, or palliation for severe brain damage.

In addition, we collected data on the long-term clinical outcome in surviving patients including diagnosis of epilepsy and functional outcome, using the Paediatric Overall Performance Category (POPC) scale performed at last follow up. The POPC is a global scale based on observer evaluation assessing the global function in daily life with the following scores: 1, normal life; 2, mild disability; 3 and 4, moderate and severe disability; 5, coma/vegetative state, and 6, brain death (22).

### 2.8. Statistical analysis

Quantitative data were presented as mean or median and interquartile range and categorical data as frequencies and percentages. The Chi-square and Fisher exact tests were used to assess the relationship among fetal and neonatal neuroimaging and cardiac ultrasound qualitative data and severe HOHF at birth requiring urgent endovascular treatment; in addition they were also used to assess the relationship among neuroradiologic and cardiologic qualitative findings with endovascular treatment complications, and demise. The Mann–Whitney *U* test was used to assess the relationship between fetal and neonatal neuroimaging and cardiac ultrasound quantitative data and the same outcome variables. Statistical analyses were performed with SPSS Statistics software, v21.0 (IBM, Armonk, NY). *P* < 0.05 was considered significant.

In the analysis of fetal variables, we included all causes of mortality. Newborns who were deemed to palliation due to severe brain damage based on prenatal assessment followed a specific clinical course and were excluded from the statistical analysis of postnatal outcomes.

## 3. Results

### 3.1. Patients

Between 2009 and 2022, 49 patients with VGAM were enrolled at Gaslini Children's Hospital. Nine patients admitted after the neonatal period were excluded. Out of the remaining 40, 34 (85%) were inborn with prenatal diagnosis. The 6 outborn patients were missed at prenatal diagnosis and were transferred to our center in the first week of life. Patients' characteristics are summarized in Table 1.

**Table 1:** Patients’ characteristics.

Birth weight, *grams*	Mean 3,115 ± 436 (range 1,920–3,630)
Gestational age, *weeks*	Mean 37.2 ± 2.10 (range 30–41)
Male	23 (57.5%)
Prenatal diagnosis *yes*	34 (85%)
Mode of delivery:	
Elective C-section	26 (65%)
Urgent C-section	6 (15%)
Vaginal delivery	8 (20%)
Apgar score at 1 min	0–9
Apgar score at 5 min	4–9
Assistance at birth	
Routine	27 (67.5%)
Positive pressure ventilation	11 (32%)
Cardiopulmonary resuscitation	2 (6%)

### 3.2. Fetal echocardiographic and neuroradiologic findings

Fetal cardiac and neuroimaging findings are summarized in Table 2.

**Table 2:** Fetal echocardiographic and neuroimaging findings and association with severe HOHF and mortality.

	All	Severe HOHF^a^	Severe HOHF^a^	*P* value	Deceased	Deceased	*P* value
		No	Yes		No	Yes	
Fetal Cardiac US	*N* = 26	*N* = 13	*N* = 10		*N* = 20	*N* = 6	
Cardiothoracic ratio, *n*	0.63 ± 0.06	0.60 ± 0.04	0.67 ± 0.07	**0.01**	0.62 ± 0.06	0.68 ± 0.04	**0.02**
Tricuspid regurgitation							
Absent	12 (46.2)	7 (53.8)	4 (40)	0.31	10 (50)	2 (33.3)	0.25
Mild	11 (42.3)	6 (46.2)	4 (40)		9 (45)	2 (33.3)	
Moderate	1 (3.8)	0	1 (10)		0	1 (16.7)	
Severe	2 (7.7)	0	1 (10)		1 (5.0)	1 (16.7)	
Reversal flow across the aortic isthmus, *yes*	20 (76.9)	8 (61.5)	9 (90)	0.18	14 (70)	6 (100)	0.28
Superior vena cava dilation, *yes*	23 (88.5)	10 (76.9)	10 (100)	0.23	17 (85)	6 (100)	1
Reversal flow in ductus venosus, *yes*	5 (19.2)	1 (7.7)	3 (30)	0.28	3 (15.0)	2 (33.3)	0.56
Fetal MRI	*N* = 24	*N* = 10	*N* = 11		*N* = 18	*N* = 6	
SS-MD, *mm*	10.09 ± 4.21	7.86 ± 3.04	11.74 ± 4.22	**0.01**	9.63 ± 4.29	11.47 ± 4.02	0.25
VGAM volume, *mm*^3^	7,924 ± 5,866	5,438.1 ± 4,317.3	8,946.5 ± 5,729.2	0.11	7,371.5 ± 5,703.0	9,582.5 ± 6,581.6	0.28
Pseudofeeders, *yes*	8 (33.3)	0	5 (45.5)	**0.03**	4 (22.2)	4 (66.7)	0.13
Ventriculomegaly, *yes*	13 (54.2)	6 (60)	4 (36.4)	0.22	10 (55.6)	3 (50)	1

HOHF, high-output heart failure; MRI, magnetic resonance imaging; SS-MD, maximal mediolateral diameter of the straight or falcine sinus at its narrowest point in the craniocaudal axis; US, ultrasound.

Bold values indicate *P* values that are considered significant.

^a^ Three patients with fetal cardiac US and MRI data were excluded from analysis of severe HOHF because deemed to palliation at birth for severe brain damage.

We could retrieve complete fetal echocardiographic data in 26 cases (65%), 10 developed severe HOHF at birth. Fetal hydrops was rare and was not included in the analysis (7%). Cardiothoracic ratio was the only finding associated with severe HOHF at birth (*P* = 0.01) and death (*P* = 0.02).

Fetal brain MR imaging was performed in 24 pregnancies (60%) at a mean gestational week at first MRI of 32.1 (range 22–37 weeks), 11 patients developed severe HOHF at birth. The mean SS-MD was 10.09 ± 4.21. Among the selected neuroradiologic variables, SS-MD and pseudofeeders showed a significant association with severe HOHF at birth (*P* = 0.01, and 0.03, respectively). None of the fetal parameters were associated with major periprocedural complications.

### 3.3. Neonatal echocardiographic and neuroradiologic findings

Neonatal findings are described in Table 3. Four patients were diagnosed with severe brain damage based on prenatal assessment, confirmed by MRI after birth, and palliative care was offered. They were excluded from the analysis. In the 36 remaining patients, echocardiographic data showed that all parameters describing right ventricular overload, pulmonary hypertension and systemic steal were significantly associated with severe HOHF requiring urgent embolization. Of note, two patients were diagnosed with sinus venosus atrial septal defect associated with partial anomalous pulmonary venous return. This association with VGAM has been previously reported in other case series (23, 24).

**Table 3:** Neonatal echocardiographic and neuroimaging findings and association with severe HOHF and mortality.

	All^a^	Severe HOHF	Severe HOHF	*P* value	Deceased	Deceased	*P* value
		No	Yes		No	Yes	
	*N* = 36	*N* = 19	*N* = 17		*N* = 33	*N* = 3	
Echocardiography							
RV dilation, *yes*	21 (58.3)	6 (31.6)	15 (88.2)	**0.001**	18 (54.5)	3 (100)	0.25
REDD, *mm*	13.76 ± 2.11	12.66 ± 2.04	15.00 ± 1.41	**≤0.0001**	13.68 ± 2.19	14.67 ± 0.58	0.41
RV *z* score	2.39 ± 0.95	1.89 ± 0.92	2.95 ± 0.61	**≤0.0001**	2.36 ± 0.98	2.78 ± 0.22	0.55
LVSF %	31.72 ± 5.28	30.95 ± 2.46	32.59 ± 7.26	0.55	31.76 ± 5.52	31.33 ± 1.15	0.59
Shape IVS							
Normal	14 (38.9)	14 (73.7)	0	**≤0.0001**	14 (43.8)	0	0.20
Intermediate	16 (44.4)	5 (26.3)	11 (64.7)		14 (42.4)	2 (66.7)	
R to L shift	6 (16.7)	0	6 (35.3)		5 (15.2)	1 (33.3)	
Shunt DA							
L to R	11 (30.6)	10 (52.6)	1 (5.9)	**≤0.0001**	11 (33.3)	0	0.25
Bidirectional	13 (36.1)	9 (47.4)	4 (23.5)		12 (36.4)	1 (33.3)	
R to L	12 (33.3)	0	12 (70.6)		10 (30.3)	2 (66.7)	
PH index							
>1	11 (30.6)	1 (5.3)	10 (58.8)	**≤0.0001**	10 (30.3)	1 (33.3)	0.95
=1	10 (27.8)	5 (26.3)	5 (29.4)		9 (27.3)	1 (33.3)	
<1	15 (41.7)	13 (68.4)	2 (11.8)		14 (42.4)	1 (33.3)	
Reversal diastolic flow at aortic isthmus, *yes*	29 (80.6)	12 (63.2)	17 (100)	**0.008**	26 (78.8)	3 (100)	1
MRI							
VGAM:							
Choroidal	30 (83.3)	13 (68.4)	17 (100)	**0.02**	27 (81.8)	3 (100)	1
Mural	6 (16.7)	6 (31.6)	0		6 (18.2)	0	
Ventriculomegaly, *yes*	12 (33.3)	4 (21.1)	8 (47.1)	**0.16**	12 (36.4)	0	0.54
SSS stenosis, *yes*	21 (58.3)	6 (31.6)	15 (88.2)	**0.001**	19 (57.6)	2 (66.7)	1
SSS index	2.68 ± 0.89	3.09 ± 0.82	2.22 ± 0.75	**0.002**	2.70 ± 0.87	2.50 ± 1.28	0.83
JB stenosis, *yes*	20 (55.6)	6 (31.6)	14 (82.4)	**0.003**	17 (51.5)	3 (100)	0.24
SS-MD, *mm*	8.26 ± 3.73	6.00 ± 2.17	10.77 ± 3.53	**≤0.0001**	8.07 ± 3.72	10.33 ± 3.88	0.22
Pseudofeeders, *yes*	14 (38.9)	2 (10.5)	12 (70.6)	**≤0.0001**	12 (36.4)	2 (66.7)	0.55

DA, ductus arteriosus; HOHF, high-output heart failure; IVS, interventricular septum; JB, jugular bulb; L, left; LVSF, left ventricular shortening fraction; PH, pulmonary hypertension; R, right; REDD, right end-diastolic diameter; RV, right ventricular; SS-MD, maximal mediolateral diameter of the straight or falcine sinus at its narrowest point in the craniocaudal axis; SSS, superior sagittal sinus.

Bold values indicate *P* values that are considered significant.

^a^ Four out of 40 patients were excluded from analysis because deemed to palliation at birth for severe brain damage.

Regarding neuroimaging data, 30 patients were classified as choroidal VGAM (83%). We found ventriculomegaly in 12 (33%), SSS stenosis in 21 (58%), JB stenosis in 20 (55.6%), and pseudofeeders in 14 (39%). The mean SS-MD was 8.26 ± 3.73. Choroidal VGAM, SSS stenosis, lower SSS index, JB stenosis, higher SS-MD, and arterial pseudofeeders were associated with severe HOHF requiring neonatal endovascular treatment. None of the neonatal variables were associated with major EVT complications and related deaths.

In Supplementary Material we provide additional data about: complete fetal and neonatal neuroradiological features in all available MRIs; occurrence of major periprocedural complications and association with fetal and neonatal echocardiographic and neuroimaging findings.

### 3.4. Intervention: medical therapy and endovascular treatment

Out of 40 newborns, 21 (52.5%) presented signs of HOHF. Seventeen (42.5%) developed severe HOHF with indication to early EVT, 19 (47.5%) were stable without support or received only medical treatment to control the signs of HOHF, and 4 (10%) were not treated due to severe brain damage at birth and received palliative care. Five patients underwent EVT for neuroradiological indications (presence of pseudofeeders, white matter lesions, or JB stenosis) and were included in the group without severe HOHF for the analysis. Data regarding intensive care support in all patients during the entire course are reported in Table 4. Invasive ventilatory support was required in 22 patients (55%), 5 of them were electively intubated before the procedure with neuroradiologic indications. Epinephrine and milrinone were mainly used for inotropic support with a maximal dose of 0.2 and 0.8 mcg/kg/min respectively, while PGE_1_ was indicated in 5 patients (12.5%). The vasoactive inotropic score, calculated on maximal doses, was 4–24 (median 10).

**Table 4:** Medical treatment and intensive care support in all newborns.

	Number of patients (%)	
Ventilatory support		
None	15 (37.5)	
Non-invasive ventilation	3 (7.5)	
Invasive ventilation	22 (55)	
Inotropes	21 (52.5)	
Epinephrin	19 (47.5)	
Milrinone	19 (47.5)	
Levosimendan	2 (5)	
PGE_1_	5 (12.5)	
Diuretics	28 (70)	
	Dose	
	Min-Max	Median of maximal dose (Q1–Q3)
Epinephrine (mcg/kg/min)	0.02–0.2	0.07 (0.04–0.09)
Milrinone (mcg/kg/min)	0.4–0.8	0.4 (0.4–0.4)
Levosimendan (mcg/kg/min)	0.1–0.2	0.15

PGE_1_, prostaglandin E_1_.

Thirty neonatal endovascular procedures were performed in 22 patients (1–3 procedures per patient) at a median age of 8 days of life (range, 2–29 days). In the severe HOHF group the first embolization was performed at a median age of 5 days of life (range, 2–15 days). Fourteen newborns did not present severe HOHF or neuroradiological indications to EVT and the therapeutic plan was deferred after discharge. All surviving patients were enrolled for clinical, cardiologic and neuroradiologic follow-up. Eleven patients underwent their first endovascular procedure at 2–6 months of life, and 3 patients never needed EVT for spontaneous VGAM reduction/closure. In the full cohort of treated patients each one underwent from 1 to 9 procedures.

**Figure 2:**
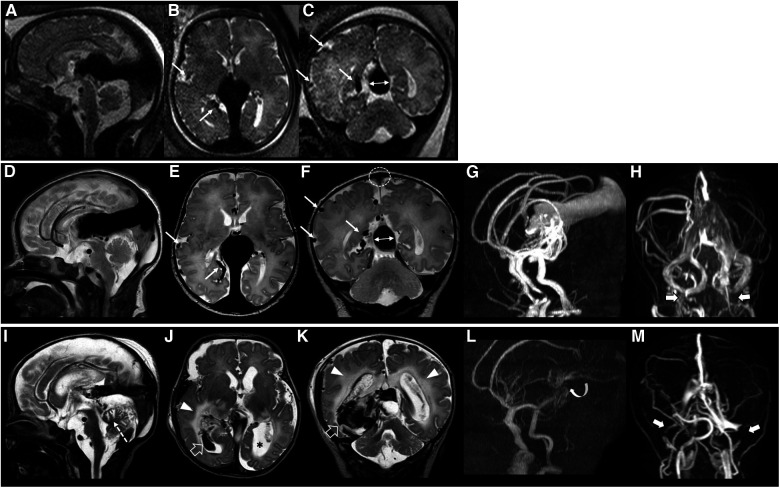
Fetal brain MRI performed at 37 gestational weeks (**A–C**), post-natal brain MRI performed at birth (**D–H**), and last brain MRI performed at 5 days of age (**I–M**), 3 days after endovascular treatment in patient #40 with a dismal prognosis. Fetal and post-natal sagittal (**A,D**), axial (**B,E**) and coronal (**C,F**) T2-weighted images demonstrate a VGAM associated with pseudofeeders from the right middle cerebral artery (thin arrows) and a high SS-MD (thin arrows with double heads, 13.5 mm). Note on post-natal MRI that there is also stenosis of the superior sagittal sinus (dotted circle) with SSS index of 2.2. MR arterial (**G**) and venous (**H**) angiography reveal a large VGAM pouch with feeders from the choroidal and anterior cerebral arteries, with stenosis of the jugular bulbs, more pronounced on the left (thick arrows). Last brain MRI (**I–K**) and MR arterial and venous angiography (**L,M**) show marked reduction of the VGAM pouch and venous efferents after embolization (curved arrow). However, there is a major complication characterized by intracranial bleeding (empty arrows), white matter edema (arrowheads) and cerebellar vermis ischemic lesion (dotted arrow). Note the ventricular dilatation, especially on the left (asterisk), and the occlusion of the transverse-sigmoid sinuses (thick arrows). The patient died soon after this last MRI.

**Figure 3:**
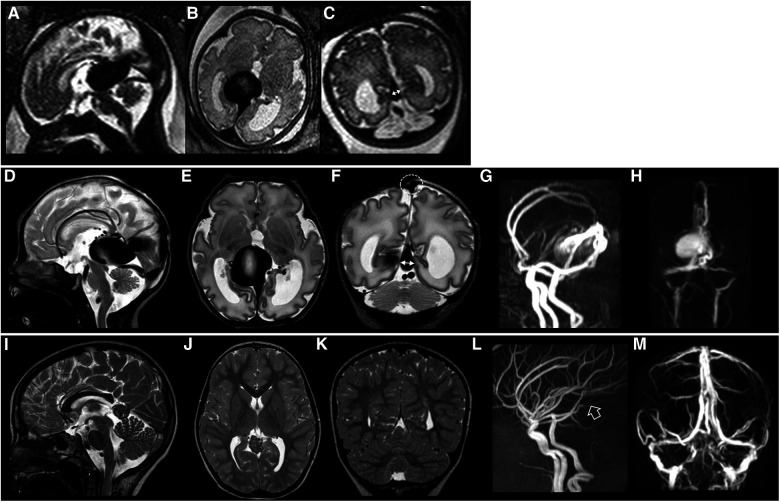
Fetal brain MRI performed at 34 gestational weeks (**A–C**), post-natal brain MRI performed at birth (**D–H**), and last brain MRI (**I–M**) performed at 8 years of age in patient #26 with a good neurological outcome. Fetal and post-natal sagittal (**A,D**), axial (**B,E**) and coronal (**C,F**) T2-weighted images demonstrate a VGAM associated with low SS-MD (thin arrows with double heads, 4.3 mm). Note on post-natal MRI that there is no stenosis of the superior sagittal sinus (dotted circle) with SSS index of 3.3. MR arterial (**G**) and venous (**H**) angiography reveal a large VGAM pouch with feeders from the choroidal and anterior cerebral arteries. There is no stenosis of the jugular bulbs. Last brain MRI (**I–K**) and MR arterial and venous angiography (**L,M**) show complete occlusion of the VGAM pouch (empty arrow). No brain complications are evident in this case.

Figure 2 illustrates the fetal and neonatal neuroimaging risk factors of HOHF, endovascular treatment complications and dismal prognosis, while Figure 3 demonstrates a case with good long-term outcome and absence of neuroimaging risk factors.

**Figure 4:**
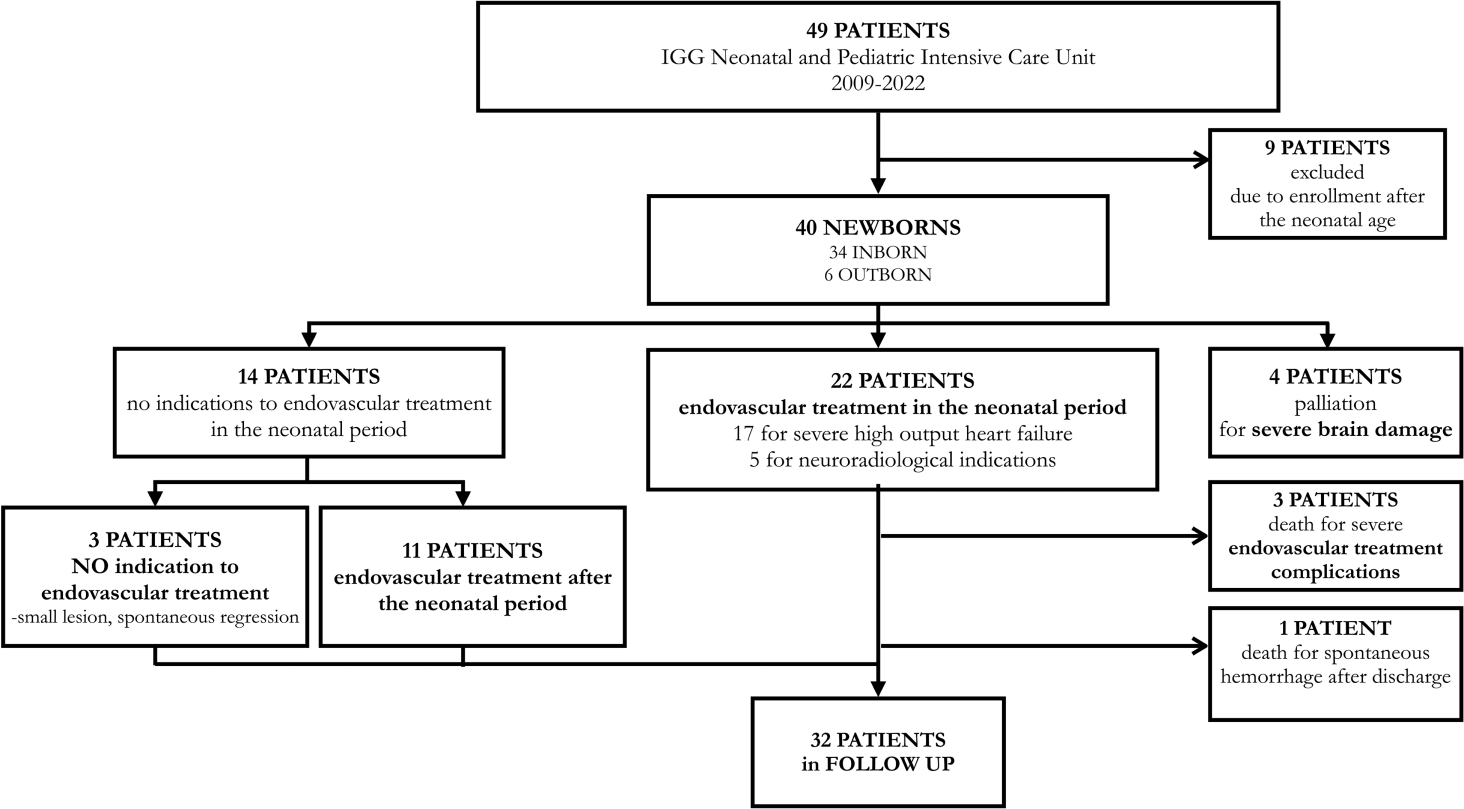
Flow diagram detailing the clinical pathways followed by the cohort of patients consecutively enrolled from 2009 to 2022 at Gaslini Children's Hospital.

The clinical course and pathways of all patients are described in Figure 4.

### 3.5. Outcomes

#### 3.5.1. Severe HOHF

**Figure 5:**
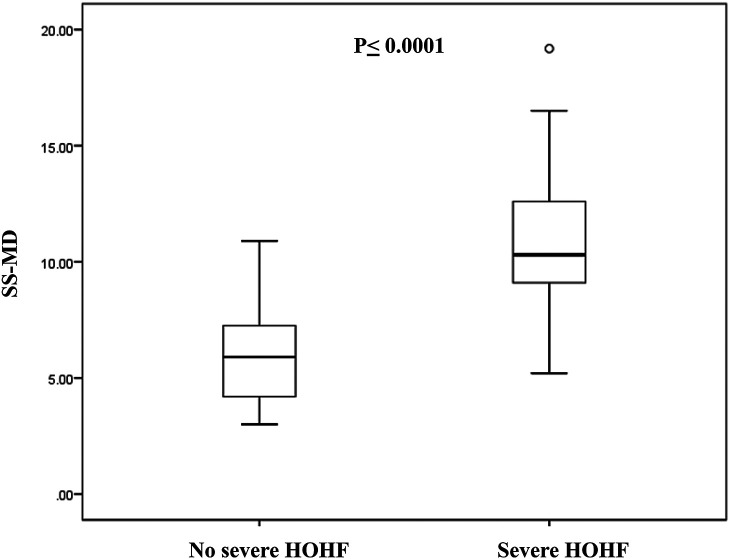
Comparison of SS-MD values on neonatal MRI in patients with severe HOHF (*N* = 17) and patients without severe HOHF (*N* = 19). The box represents the interquartile range (25–75th percentile) of the 2 populations and the line within this box is the median value. Bottom and top bars of the whisker indicate the 10th and 90th percentiles, respectively.

As previously mentioned, 17/40 patients (42.5%) developed severe HOHF with indication to early EVT. These patients presented higher fetal CTR (0.67 ± 0.07), neonatal echocardiographic features of RV dilation, shift of the IVS, PH ≥ 1, bidirectional or right to left shunt in DA, and reversal diastolic flow at the aortic isthmus. The neuroradiologic findings characterizing this group of newborns were higher SS-MD (fetal 11.74 ± 4.22, neonatal, 10.77 ± 3.53), presence of pseudofeeders, SSS stenosis, and JB stenosis; they were classified as choroidal VGAM in 100% of cases. Comparison of SS-MD values on neonatal MRI in patients with severe HOHF and without severe HOHF is showed in Figure 5.

#### 3.5.2. Periprocedural complications

Periprocedural complications occurred in 10/22 newborns (45%), 4 (18%) were defined as minor and 6 (27%) as major. Two patients required ventriculoperitoneal shunt placement. Of note, in the cohort of 11 patients treated after the neonatal period only one patient (9%) experienced a periprocedural complication, defined as major.

#### 3.5.3. Mortality

Death occurred in 7/40 patients (18%) in the neonatal period. Indication to palliation for congenital severe brain damage was deemed in 4/40 newborns (10%), and 3 died after the occurrence of severe periprocedural complications (14% of 22 treated patients). None of the patients died due to HOHF and multiorgan failure. We found that there was no association of VIS values, as index of severity of HOHF, with occurrence of EVT complications (*P* = 0.27) and death (*P* = 0.13). In this cohort one patient deceased after discharge, 1 month after the endovascular procedure for spontaneous intracranial hemorrhage.

#### 3.5.4. Follow up

Follow-up was completed in 100% of surviving patients (32/40), with a median duration of 5.1 years (range 7 months to 13 years). Poor neurological outcome and epilepsy were observed in 8 patients (25%). At last follow-up, the POPC scale was defined as 1 in 23 subjects (72%), 2 in 1 case (3%), 3 in 5 patients (16%), and 4 in the remaining 3 cases (9%).

## 4. Discussion

This study on a cohort of 40 newborns provides insights in the complex medical and interventional management of VGAM from prenatal diagnosis to long-term follow up. Assessment and treatment planning, especially in the perinatal period, have a key role in defining the clinical course and outcomes of this disease. In the past the neonatal mortality was equal to 100% in the most severe forms. Despite the advances in neuroendovascular techniques a high rate of mortality and severe neurodevelopmental morbidity among survivors is still reported (9).

Our experience provides relevant data and presents a paradigm to guide clinical strategies, in terms of medical treatment, and indication and timing of endovascular procedures. The potential effects of aggressive intensive care support on disease course and outcomes, following the correct interpretation of VGAM physiopathology, are outlined in this study.

In the first place, we aimed to evaluate echocardiographic and neuroradiologic factors obtained from fetal and early neonatal studies that may represent predictors of clinical presentation and short term outcomes in the neonatal period, including severe HOHF, periprocedural complications and death.

The role of fetal echocardiography in VGAM prenatal evaluation is well-known for the assessment of cardiomegaly, and other signs of HF progression and for excluding possible associated morphological cardiac abnormalities. Cardiomegaly and tricuspid regurgitation seem to be relevant to anticipate the level of haemodynamic impairment at birth and have been related to prognosis (2, 12, 25). In our cohort although tricuspid regurgitation, dilation of SVC, and reversal diastolic flow across the aortic isthmus were frequent findings in newborns with heart failure, only fetal cardiothoracic ratio was associated with severe HOHF and mortality.

As expected, in newborns all parameters that are expression of right heart overload and pulmonary overflow and hypertension were significantly associated with severe HOHF. Moreover, the right to left shunt in the DA and the reversal diastolic flow across the aortic isthmus describes the effects of the massive AV shunt on the pulmonary and systemic circulation, potentially leading to multiorgan failure without intervention. Echocardiographic data were useful to support decision making, but the need for invasive ventilation and inotropic support were the main determinants of endovascular treatment. Medical treatment may grant stabilization of the clinical status even in patients with echocardiographic signs of right heart overload and pulmonary hypertension and allow deferral of the procedure after the neonatal period.

Regarding MRI findings, recent reports on fetal and neonatal prognostic factors highlighted the role of SS-MD (9), and arterial pseudofeeders (10, 26, 27). Our study confirms that these parameters predict severe HOHF at birth, and, on fetal evaluation they were the only significant factors in this cohort. Arko et al. studied SS-MD and demonstrated that dilation at this point was sharply predictive of neonatal decompensation necessitating endovascular intervention, in both fetal and neonatal MR imaging (9). This parameter may allow to define risk stratification and timing of treatment in newborns, as well as to identify potential candidates for novel interventions, such as fetal embolization (28). If we consider the SS-MD mean values obtained in our study in patients with severe HOHF (fetal 11.74 mm, and neonatal 10.77 mm) the probability determined by logistic regression of clinical evolution to neonatal at risk cohort, according to Arko at al., would have been 96%–98%. Furthermore, fetal pseudofeeders have been reported as risk factors of severe cardiac failure, pulmonary hypertension, and encephalomalacia at birth (10). It is important to note that, in the absence of obvious fetal brain damage, they were associated with poor brain outcomes at birth in approximately half of the newborns (10), and early after birth (26). This evidence led to consider pseudofeeders as indication to early delivery for emergency embolization, as well as indication to emergency treatment after birth if present on neonatal MRI (29). In our series arterial pseudofeeders were identified in all fetal and 70% of neonatal studies of patients who developed severe HOHF, proving their relevance in identifying critical cases.

On neonatal MRI, other findings were associated with severe HOHF in our cohort, including: choroidal type VGAM, SSS stenosis, and JB stenosis. As observed by Lasjaunias et al., choroidal-type VGAMs are more common in the neonatal presentation and are more likely to produce medically untreatable HOHF (21). This is confirmed by our experience, where all patients with severe HOHF had choroidal VGAM. The venous abnormalities observed in patients with VGAM are frequent and are thought to be related to retention of fetal anatomical features and inhibition of proper maturation of the jugular bulb and sigmoid sinus due to the AV fistula (1). Saliou et al. showed that a decreased diameter of the SSS at presentation or progressive JB narrowing or occlusion are associated with poor clinical outcome. According to the authors the first may reflect a decrease of the cerebral blood flow due to cerebral arterial steal and intracranial hydrovenous disorders, and the second may be related to abnormal skull maturation, or venous high flow angiopathy. If the JB stenosis is severe, venous congestion may lead to chronic venous ischemia, delayed calcifications, and the so-called “pseudophlebitic” appearance of the cortical veins (17). Both findings may indicate early intervention to prevent progressive damage. It will be relevant to study the association of the described venous abnormalities with long term outcomes in our cohort.

In terms of neurologic outcome, it is well-known that major hemorrhagic and ischemic complications related to endovascular treatment, especially in the neonatal period, strongly impact the neurological outcome of these children. In our study, almost one-third of treated cases had a major complication, highlighting the high risk of EVT in this critical period. Contributing factors to major neurological complications after EVT have been described, including internal cerebral vein drainage pattern, large diameters of microcatheters, excessive single-session embolization, and others (30). We could not find any echocardiographic or neuroradiologic variable that was associated with EVT complications. A focused analysis of specific procedural risk factors should be the topic of further research in this case series. In our cohort, all patients who experienced major EVT complications developed poor neurological outcome, epilepsy, or died. On the other hand, the majority of surviving patients (75%) presents normal outcome or mild overall disabilities (POPC score of 1 or 2), including patients presenting with severe HOHF in the neonatal period. This further highlights the value of each step of the diagnostic and therapeutic pathway that we presented.

A keypoint in intensive care support and haemodynamic management is the clear understanding of the physiopathology of the disease. Strategies are not well defined or at least controversial, considering the conflicting therapeutic approaches that have been described in published case series. Recommendations based on review of the literature and experience of the authors at their own institutions have been recently published and reflect the modern approach to HOHF in VGAM (6). The clinical effects of the high flow-low resistance cerebral shunt may present with minimal signs of cardiac overload or, at the end of the spectrum, with cardiogenic shock with arterial hypoxemia and acidosis. The haemodynamic features and echocardiographic findings related to VGAM are illustrated in Supplementary Figure S1.

The objective of medical treatment in newborns with VGAM should be to reduce the main components of HOHF by providing inotropic support and peripheral vasodilation. The optimal choice of vasoactive medications is not standardized (6). In our unit we mainly use epinephrin and milrinone as they convey, in combination, predictable effects, and can be easily titrated. The VIS values that we report, calculated on maximal doses, may seem low compared to previous studies (20). This is likely due the to the fact that we only use epinephrine and milrinone among the medications included in the VIS score. A standard epinephrine dose of 0.04 mcg/kg/min and milrinone of 0.4 mcg/kg/min will lead to a VIS score of 8. Levosimendan is a calcium-sensitizing agent with unique properties as it combines inotropic and vasodilating effects, without increasing heart rate and myocardial oxygen consumption (6, 31). Our experience, so far, is limited, but levosimendan appears to be a promising therapeutic resource in this specific haemodynamic setting, and may deserve further investigation. Although in many centers pulmonary vasodilators still have a central role in VGAM treatment, they should be avoided in the first few days of life since they magnify the negative effects of pulmonary overflow (altered gas exchange, compromise of pulmonary vessels maturation, pulmonary hypertension) and worsen the physiopathological mechanisms of HF. None of our patients were discharged on pulmonary vasodilators, demonstrating that fetal/neonatal pulmonary maturation is not compromised, and significant vascular remodeling does not occur with this approach. Mechanical ventilation is often required to reduce cardiac workload and guarantee oxygenation and ventilation. PGE_1_ may be indicated to keep the ductus arteriosus open to treat the right ventricular overload and prevent myocardial ischemia by reducing the transmural pressure gradient in most severe cases. With this strategy medical management improved heart failure, optimized systemic circulation and allowed the endovascular procedure in stable conditions. In our experience and in this case series cardiac and/or respiratory failure refractory to medical treatment leading to multiorgan failure never occurred. Moreover, we demonstrated that patients requiring maximal inotropic support, expressed by the vasoactive inotropic score, had no increased risk of EVT complications and/or death. This is the reason we consider the Bicêtre neonatal evaluation score no longer relevant to define indication to treatment or palliation. The Bicêtre score was created to facilitate treatment decision and triage newborns into therapeutic abstention, urgent or deferred endovascular treatment (21). The essential fallout of this strategy is that severe HOHF never represented an indication to palliation in our center. This may explain the low neonatal mortality due to palliation we reported (10%) compared to previous series, where mortality due to palliation accounted for 18%–42% of cases with brain damage and/or MOF (20, 30, 32–35). Considering neonatal overall mortality, we report a 18% combined mortality due to palliation and endovascular procedure complications. This rate is lower compared to historical data, 52% in Lasjaunias et al. publication (21), and to more recent large series and meta-analysis reporting a neonatal mortality of 31%–63% (20, 30, 32–36), although it is difficult to draw a reliable conclusion on this matter.

The main limitations of the study are the retrospective design and the small sample size of this case series. VGAM is a rare disorder, and the size of this cohort is similar to the ones previously published by other referral centers (30, 32, 33, 35). Fetal data are not available for all patients, but this is a common limitation when dealing with fetal studies (9, 10, 25) and this is also related to the possibility of postnatal diagnosis and referral. We should underline that our positive results in mortality and outcomes are not influenced by a selection bias. This is a cohort of consecutive patients, none of which has been excluded for incomplete data or missing follow up. On the contrary, we excluded from the analysis the nine patients that were enrolled in our center beyond the neonatal age. It has been recognized that patients presenting after the newborn period have more favorable neurological outcomes (37). This cohort includes all cases of prenatal diagnosis referred to our center. We do not report any case of termination of pregnancy due to diagnosis of VGAM, that may have determined a selection bias in this population. We decided to exclude from the postnatal analysis the four newborns on palliative care for congenital severe brain damage, although they all presented with severe HOHF. Their support and clinical course were influenced by the prognostic indication.

Among the other limitations, the data obtained from brain MRI and MRA were not always confirmed by angiography, that was performed in newborns only for therapeutic purposes. Indeed, technical limitations related to the contrast dose did not allow further diagnostic studies during angiography.

Another limitation is related to the assessment of neurological outcome with the POPC scale, that represents a gross functional evaluation, mainly based on motor deficits. It is well-known that in VGAM patients neurocognitive difficulties may emerge with age and are mainly related to the chronic effect of vascular steal and intracranial venous hypertension. A standardized and comprehensive neurocognitive assessment protocol has been recently implemented in our center for VGAM patients. The results of the prospective evaluations in this cohort will be the objective of a forthcoming study, with more precise and valuable outcome measures.

## 5. Conclusions

Few centers in the world deal with the management of patients with VGAM, from prenatal diagnosis to long term follow up. Diagnostic definition and decision-making are still very challenging. Fetal and neonatal echocardiographic and neuroradiologic findings are essential for exploring the best strategy for delivery, for accurate risk stratification of newborns, and for family counseling at prenatal diagnosis and afterwards. Outcomes may be improved with clinical strategies based on a multidisciplinary approach, specialized intensive care support focused on VGAM physiopathology, and accurate indication and timing of endovascular treatments.

In rare diseases it is difficult to draw meaningful conclusions based on case series. Therefore, international collaboration and a multicenter registry should be the foundation of future research, to improve the knowledge on this complex disease, and to make real progress in the outcomes and quality of life of children with VGAM.

## Data Availability

The original contributions presented in the study are included in the article/**Supplementary Material**, further inquiries can be directed to the corresponding author.

## Supplementary material

Supplementary Figure S1 Physiopathology of high output heart failure secondary to high flow AV shunt in vein of Galen aneurysmal malformation. Haemodynamic features and related echocardiographic effects are illustrated and described.
