# Supplementary material for: Vein of Galen aneurysmal malformation in newborns: a retrospective study to describe a paradigm of treatment and identify risk factors of adverse outcome in a referral center
Source: Front Pediatr. 2023 Jul 20;11:1193738. doi: 10.3389/fped.2023.1193738 (PMC10426803; doi:10.3389/fped.2023.1193738)
Supplement: Supplementary file 1 [file Table1.docx]

**Table. Fetal and neonatal neuroradiological features**

| **N (%)** | **Fetal MRI (n=24)** | **Neonatal MRI (n=39)** |
| --- | --- | --- |
| Choroidal | -- | 33 (92%) |
| Ventriculomegaly | 13 (54.1%) | 14 (35.8%) |
| Global brain atrophy | 6 (25%) | 3 (7.6%) |
| Ischemic-hemorrhagic lesions | 4 (16.6%) | 13 (33.3%) |
| WM lesions | 8 (33.3%) | 18 (46.1%) |
| Pseudofeeders | 8 (33.3%) | 17 (43.5%) |
| Aqueductal compression | 16 (66.6%) | 25 (64.1%) |
| SSS stenosis | NA | 24 (61.5%) |
| JB stenosis | NA | 23 (58.9%) |
| ICV drainage into VGAM | NA | 8 (20.5%) |
| Small thalamic feeders | NA | 13 (33.3%) |

**Legend:** ICV, internal cerebral vein; JB, jugular bulb; NA, not assessable; MRI, magnetic resonance imaging; SSS, superior sagittal sinus; VGAM, WM, white matter.
