# Supplementary material for: Vein of Galen aneurysmal malformation in newborns: a retrospective study to describe a paradigm of treatment and identify risk factors of adverse outcome in a referral center
Source: Front Pediatr. 2023 Jul 20;11:1193738. doi: 10.3389/fped.2023.1193738 (PMC10426803; doi:10.3389/fped.2023.1193738)
Supplement: Supplementary file 2 [file Table2.docx]

**Table. Fetal echocardiographic and neuroradiologic variables and association with EVT complications.**

|  | All |  | EVT complications  no | EVT complications  yes | P value |
| --- | --- | --- | --- | --- | --- |
| Fetal Cardiac US | N=19 |  | N=14 | N=5 |  |
|  |  |  |  |  |  |
| Cardiothoracic ratio | 0.63±0.06 |  | 0.62±0.07 | 0.67±0.05 | 0.13 |
| Tricuspid regurgitation absent | 8 (42.1) |  | 5 (35.7) | 3 (60) | 0.62 |
| mild | 9 (47.4) |  | 7 (50) | 2 (40) |  |
| moderate | 1 (5.3) |  | 1 (7.1) | 0 |  |
| severe | 1 (5.3) |  | 1 (7.1) | 0 |  |
| Reversal flow across the aortic isthmus, *yes* | 14 (73.7) |  | 9 (64.3) | 5 (100) | 0.26 |
| Superior vena cava dilation, *yes* | 16 (84.2) |  | 11 (78.6) | 5 (100) | 0.53 |
| Reversal flow in ductus venosus, *yes* | 4 (21.1) |  | 2 (14.3) | 2 (40) | 0.27 |
|  |  |  |  |  |  |
| Fetal MRI | N=19 |  | N=11 | N=8 |  |
|  |  |  |  |  |  |
| SS-MD*, mm* | 10.26±4.17 |  | 10.44±5.28 | 10.02±2.18 | 0.49 |
| VGAM volume*, mm^3^* | 7733.1±5352.8 |  | 8318.1±6376.1 | 6928.9±3784.3 | 0.90 |
| Pseudofeeders, *yes* | 5 (26.3) |  | 3 (27.3) | 2 (25) | 1 |
| Ventriculomegaly, *yes* | 10 (52.6) |  | 8 (72.7) | 2 (25) | 0.07 |

**Legend**: EVT: endovascular treatment; MRI: magnetic resonance imaging; SS-MD: maximal mediolateral diameter of the straight or falcine sinus at its narrowest point in the craniocaudal axis; US: ultrasound.
