# Supplementary material for: Vein of Galen aneurysmal malformation in newborns: a retrospective study to describe a paradigm of treatment and identify risk factors of adverse outcome in a referral center
Source: Front Pediatr. 2023 Jul 20;11:1193738. doi: 10.3389/fped.2023.1193738 (PMC10426803; doi:10.3389/fped.2023.1193738)
Supplement: Supplementary file 3 [file Table3.docx]

**Table. Neonatal echocardiographic and neuroradiologic variables and association with EVT complications.**

|  | **All** |  | **EVT complications**  **no** | **EVT complications**  **yes** | **P value** |
| --- | --- | --- | --- | --- | --- |
|  | N=22 |  | N=14 | N=8 |  |
| **Echocardiography** |  |  |  |  |  |
|  |  |  |  |  |  |
| RV dilation, *yes* | 16 (72.7) |  | 9 (64.3) | 7 (87.5) | 0.35 |
| REDD, *mm* | 14.27±2.07 |  | 14.14±2.51 | 14.50±1.07 | 0.71 |
| RV z score | 2.63±0.92 |  | 2.56±1.10 | 2.74±0.49 | 0.76 |
| LVSF % | 32.36±6.57 |  | 32.93±8.19 | 31.38±1.77 | 0.71 |
| Shape IVS normal | 2 (9.1) |  | 2 (14.3) | 0 | 0.32 |
| intermediate | 14 (63.6) |  | 9 (63.4) | 5 (62.5) |  |
| R to L shift | 6 (27.3) |  | 3 (21.4) | 3 (37.5) |  |
| Shunt DA L to R | 4 (18.2) |  | 3 (21.4) | 1 (12.5) | 0.82 |
| bidirectional | 6 (27.3) |  | 4 (28.6) | 2 (25) |  |
| R to L | 12 (54.5) |  | 7 (50) | 5 (62.5) |  |
| PH index >1 | 11 (50) |  | 6 (42.9) | 5 (62.57) | 0.28 |
| =1 | 5 (22.7) |  | 4 (28.6) | 1 (12.50) |  |
| <1 | 6 (27.3) |  | 4 (28.6) | 2 (25) |  |
| Reversal diastolic flow at aortic isthmus, *yes* | 20 (90.9) |  | 12 (85.7) | 8 (100) | 0.51 |
|  |  |  |  |  |  |
| **MRI** |  |  |  |  |  |
|  |  |  |  |  |  |
| VGAM: Choroidal | 19 (86.4) |  | 11 (78.6) | 8 (100) | 0.27 |
| Mural | 3 (13.6) |  | 3 (21.4) | 0 |  |
| Ventriculomegaly, *yes* | 10 (45.5) |  | 8 (57.1) | 2 (25) | 0.20 |
| SSS stenosis, *yes* | 16 (72.7) |  | 9 (64.3) | 7 (87.5) | 0.35 |
| SSS index | 2.50±0.94 |  | 2.64±1.00 | 2.25±0.83 | 0.48 |
| JB stenosis, *yes* | 17 (77.3) |  | 9 (64.3) | 8 (100) | 0.11 |
| SS-MD, *mm* | 9.69±3.77 |  | 9.38±4.28 | 10.24±2.85 | 0.30 |
| Pseudofeeders, *yes* | 13 (59.1) |  | 7 (50) | 6 (75) | 0.38 |

**Legend:** DA: ductus arteriosus; EVT: endovascular treatment; HOHF: high-output heart failure; IVS: interventricular septum; JB: jugular bulb; L: left; LVSF: left ventricular shortening fraction; PH: pulmonary hypertension; R: right; REDD: right end-diastolic diameter; RV: right ventricular; SS-MD: maximal mediolateral diameter of the straight or falcine sinus at its narrowest point in the craniocaudal axis; SSS: superior sagittal sinus.
